# A Novel Mock Circuit to Test Full-Flow Extracorporeal Membrane Oxygenation

**DOI:** 10.3390/membranes12050493

**Published:** 2022-04-30

**Authors:** Stefan Caspari, Leonie S. Schwärzel, Anna M. Jungmann, Nicole Schmoll, Frederik Seiler, Ralf M. Muellenbach, Marcin Krawczyk, Quoc Thai Dinh, Robert Bals, Philipp M. Lepper, Albert J. Omlor

**Affiliations:** 1 Department of Internal Medicine V—Pneumology, Allergology and Intensive Care Medicine, University Hospital of Saarland, 66424 Homburg, Germany; stefancaspari@gmx.de (S.C.); leonie.schwaerzel@gmx.de (L.S.S.); a.jungmann@t-online.de (A.M.J.); ni-schmi@gmx.de (N.S.); frederik.seiler@uks.eu (F.S.); thai.dinh@uks.eu (Q.T.D.); robert.bals@uks.eu (R.B.); albert.omlor@uks.eu (A.J.O.); 2 Department of Anaesthesiology and Critical Care, Campus Kassel of the University of Southampton, 34125 Kassel, Germany; ralf.muellenbach@klinikum-kassel.de; 3 Department of Internal Medicine II, University Hospital of Saarland, 66424 Homburg, Germany; marcin.krawczyk@uks.eu; 4 Laboratory of Metabolic Liver Diseases, Department of General, Transplant and Liver Surgery, Centre for Preclinical Research, Medical University of Warsaw, 02091 Warsaw, Poland

**Keywords:** ECMO, COPD, ARDS, mock circulation, recirculation loop

## Abstract

Extracorporeal membrane oxygenation (ECMO) has become an important therapeutic approach in the COVID-19 pandemic. The development and research in this field strongly relies on animal models; however, efforts are being made to find alternatives. In this work, we present a new mock circuit for ECMO that allows measurements of the oxygen transfer rate of a membrane lung at full ECMO blood flow. The mock utilizes a large reservoir of heparinized porcine blood to measure the oxygen transfer rate of the membrane lung in a single passage. The oxygen transfer rate is calculated from blood flow, hemoglobin value, venous saturation, and post-membrane arterial oxygen pressure. Before the next measuring sequence, the blood is regenerated to a venous condition with a sweep gas of nitrogen and carbon dioxide. The presented mock was applied to investigate the effect of a recirculation loop on the oxygen transfer rate of an ECMO setup. The recirculation loop caused a significant increase in post-membrane arterial oxygen pressure (paO_2_). The effect was strongest for the highest recirculation flow. This was attributed to a smaller boundary layer on gas fibers due to the increased blood velocity. However, the increase in paO_2_ did not translate to significant increases in the oxygen transfer rate because of the minor significance of physically dissolved oxygen for gas transfer. In conclusion, our results regarding a new ECMO mock setup demonstrate that recirculation loops can improve ECMO performance, but not enough to be clinically relevant.

## 1. Introduction

Extracorporeal membrane oxygenation (ECMO) is a powerful method to oxygenate blood and to deplete it from carbon dioxide [1]. It is used in patients with severe acute respiratory distress syndrome (ARDS), and also became an important therapeutic approach in the COVID-19 pandemic [1,2,3].

The development and establishment of new ECMO systems relies on expensive in vivo testing using animal models [4,5]. In vitro test systems (mock circuits) for ECMO present a reasonable and interesting alternative for ECMO research. Few mock circuits for ECMO are already described in the literature [4,6,7,8,9]. However, until now, real-time measurement of the oxygen transfer rate of a membrane lung in a mock model has not been described in the literature.

In general, there are three types of mock models for extracorporeal lung support: continuous measurement systems with a closed-loop system or with a dual-loop system, both mainly used for simulation of extracorporeal carbon dioxide removal (ECCO_2_R) and discontinuous measurement systems [6,10,11,12,13]. CO_2_ removal of a membrane lung is mainly determined by the sweep gas flow and can be sufficiently performed at low blood flow rates [14]. In our previous publications [13,14], we demonstrated ECCO_2_R mocks that consisted of a test circuit connected to another circuit with another membrane lung, the primary circuit. The membrane lung in the primary circuit can continuously remove oxygen and add CO_2_ with a sweep gas of nitrogen and CO_2_. As a result, venous blood for the ECCO_2_R in the test circuit was continuously supplied. However, this approach is not sufficient for an ECMO model with its higher blood flow [15] and oxygen intake because the ability to remove oxygen in those mocks is too low. In fact, it is much harder for a membrane lung to remove oxygen from blood than to add it. The reason for this is the higher diffusion gradient for O_2_ from pure oxygen gas to venous blood compared to the gradient for O_2_ from arterial blood to a nitrogen carbon dioxide gas mixture. In the mentioned ECCO_2_R mock, if the blood flow in the test circuit exceeded 1.5 L/min, the oxygen-removing membrane would be unable to remove that much oxygen, no matter how high the N_2_ sweep gas rates were set. As a result, the saturation of the blood at the intake of the ECCO_2_R or ECMO to be tested would rise to a point where it could no longer be called venous.

To circumvent this problem, we developed a completely new approach compared to the dual-loop mocks. Unlike our ECCO_2_R mocks that were focused on measuring a CO_2_ transfer rate, our new ECMO mock was designed to measure the O_2_ transfer rate. We therefore had to develop a convenient way to measure the oxygen transfer rate in real-time in order to evaluate the oxygenation efficiency of membrane lungs under different conditions.

## 2. Materials and Methods

### 2.1. Design of the ECMO Mock Model

**Figure 1:**
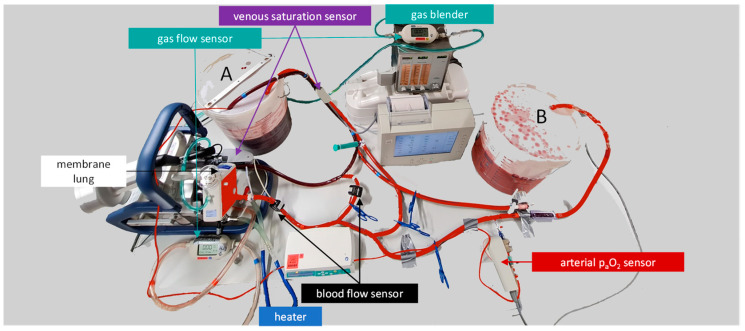
Schematic layout of the mock circuit. The mock consists of two buckets (A and B) as reservoirs, a Quadrox iR adult membrane lung with an integrated pump, and 3/8” polyvinyl chloride tubings. In addition to the venous saturation sensor of the Cardiohelp platform that drives the membrane lung, a CDI Blood Parameter Monitoring System 550 adds a second venous saturation and hemoglobin sensor, as well as an arterial side-flow sensor for the oxygen pressure (paO_2_). The mock can be used to test the oxygen transfer over a membrane lung.

The presented ECMO mock model uses two 12-liter (L) buckets, a Quadrox-iR adult 7 L membrane lung with an integrated centrifugal pump, and 3/8″ polyvinyl chloride tubings. The system is driven by the Getinge Cardiohelp platform. The system is tempered to 37 °C with a Maquet HU 35 via the membrane lung (Figure 1). The path that the blood takes can be modified by clamping certain tubings. Initially, bucket A is filled with approximately 12 L of heparinized fresh porcine blood as the test fluid. The 12 L of blood, a mix of blood from multiple pigs, is added with 10.000 IE/L of heparin to avoid clotting and 1g of meropenem to avoid bacterial growth during the measurement.

**Figure 2:**
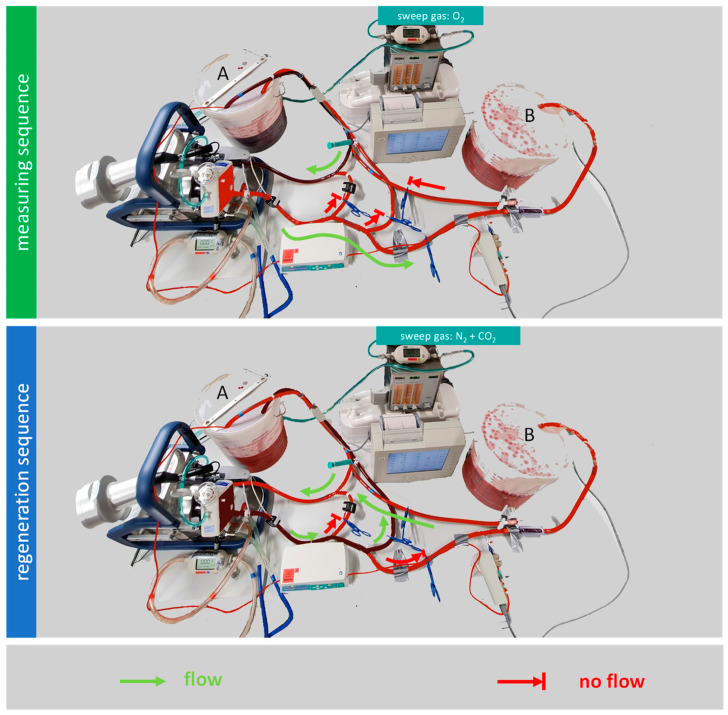
The mock measures discontinuously. During the measuring sequence, the venous blood in bucket A is oxygenated by the membrane lung, and then pumped into bucket B. After the measuring sequence, the arterial blood in bucket B is transferred to bucket A and regenerated to venous blood. During this regeneration sequence, the blood circulates only between bucket A and the membrane lung. In the regeneration sequence, a sweep gas of nitrogen and carbon dioxide is applied to the membrane lung.

The model operates in two sequences: alternating regeneration sequences and measuring sequences (Figure 2).

#### 2.1.1. Regeneration Sequence

Before each measurement, blood must be converted to a venous environment, which is termed “the regeneration sequence”. Oxygen is removed and CO_2_ is added to the porcine blood by circulating it from bucket A to the membrane lung, and then back to bucket A. In order to achieve the oxygen removal, the sweep gas composition in the gas blender is set to a mixture of N_2_ and CO_2_. A sweep gas flow of 8.5 L/min N_2_ and 0.7 L/min CO_2_ was chosen to achieve a carbon dioxide partial pressure (pCO_2_) of 40 mmHg ± 5 mmHg. The sweep gas flow is stopped once the desired saturation at the venous sensor is reached, which in this case is 65%.

#### 2.1.2. Measuring Sequence

When the blood in bucket A has reached a venous saturation of 65%, the measuring sequence can begin. The blood flow that leaves the membrane lung is redirected from bucket A to bucket B. The blood now flows from bucket A to the membrane lung, and then to bucket B. During the measuring sequence, the membrane lung receives pure oxygen as sweep gas from the gas blender. In this experiment, the sweep gas flow was set to 7 L/min using a mass flow sensor (TSI41403). During the measuring sequence, the oxygen transfer can be calculated from the blood flow, the hemoglobin value, the venous oxygen saturation before the membrane lung, and the arterial oxygen pressure behind the membrane lung. Those values are measured with the onboard sensors of the Cardiohelp and with a separate CDI Blood Parameter Monitoring System 550, which has a venous sensor and an arterial side flow sensor. The terms venous and arterial are not related to the actual placement of a cannula here; they are used as synonyms for blood leaving the patient (= venous) and blood getting back to the patient (= arterial). The flow over the arterial side flow sensor is ensured by a Braun Infusomat Space. Six repeated measurements are taken for each data point. The oxygen transfer over the membrane lung is calculated as

Q_va_O_2_ = Q_blood_ [dL/min] × (Hb [g/dL] × 1.34 [mL/g] × S_a_O_2_ + p_a_O_2_ [mmHg] × 0.0031 [1/mmHg × mL/dL] − Hb [g/dL] × 1.34 [mL/g] × S_v_O_2_)

At the end of the measuring sequence, bucket A is empty and bucket B is filled with oxygen-rich and CO_2_-depleted arterial blood. Before the next measurement, the blood must be transferred to bucket A, and another regeneration sequence has to take place. All measurements were carried out on the same day with the same batch of blood.

### 2.2. Testing the Effect of a Recirculation Loop on the Oxygen Transfer Rate

**Figure 3:**
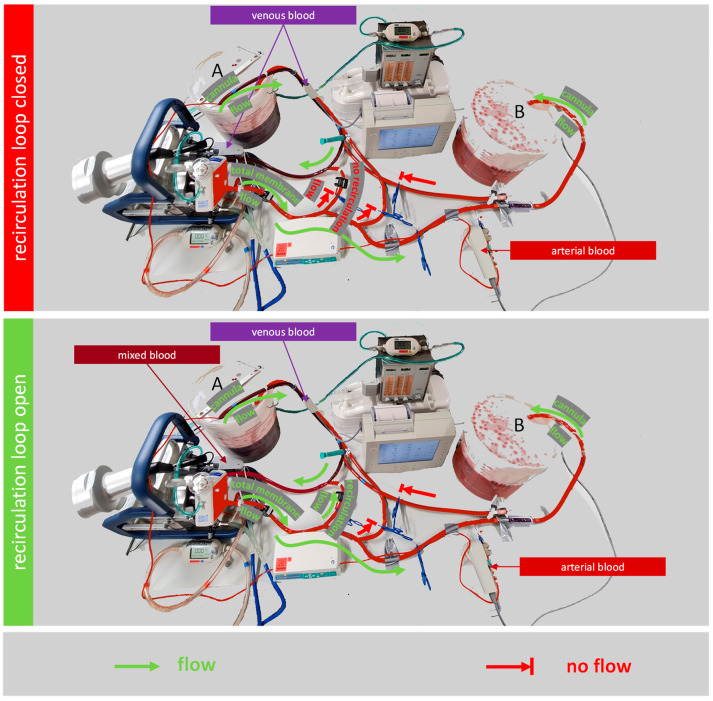
Test setup to measure the effect of a recirculation loop on the oxygen transfer rate of the membrane lung. Both schematic illustrations show the mock circuit during the measuring sequence. In the upper illustration, the recirculation loop is closed. The membrane lung has venous blood as input. In the lower illustration, the recirculation loop is open and transfers arterial blood that already passed the membrane lung back to the inlet of the pump. The membrane lung has mixed blood as input. In both setups, the oxygen transfer rate of the membrane lungs is calculated from the cannula blood flow, the hemoglobin value, the venous saturation (bucket A), and the arterial oxygen pressure (bucket B).

In this study, the ECMO model is used to assess the effect of a recirculation loop on the oxygenation efficiency of a membrane lung. For this, a direct connection from the outlet of the membrane lung to the inlet of the centrifugal pump, the recirculation loop, was added to the mock circuit (Figure 3). The recirculation loop has an individual blood flow sensor and can be throttled to a given blood flow. The blood flow that leaves or enters the buckets is called cannula flow in analogy to a clinical ECMO setup. The cannula flow is not measured, but calculated as the difference of the total membrane flow, which is measured at the membrane lung outlet, minus the recirculation flow, which is measured at the recirculation loop.

Three configurations were compared in the following settings: (a) 2.5 L/min total membrane flow, 2.5 L/min cannula flow, 0 L/min recirculation flow; (b) 3.5 L/min total membrane flow, 2.5 L/min cannula flow, 1 L/min recirculation flow; and (c) 7 L/min total membrane flow, 2.5 L/min cannula flow, 4.5 L/min recirculation flow.

### 2.3. Statistics

Statistical analysis was performed with a GraphPad Prism 5.02 (GraphPad Software, Inc., La Jolla, CA, USA). Data were presented as means ± SEM. The Kolmogorov–Smirnov test was used to evaluate the distribution of the tested values. Differences between groups were analyzed using a Bonferroni post-test with one-way ANOVA, where *p*-values of <0.05 (*) and <0.01 (**) were considered significant, and *p*-values < 0.001 (***) were considered highly significant.

## 3. Results

### 3.1. Design of the ECMO Mock Model

We were able to create a realistic model with our mock circuit with a venous saturation of 65% and a post-membrane arterial oxygen pressure (p_a_O_2_) between 400 mmHg and 500 mmHg. With a blood volume of 12 L and a cannula flow of 2.5 L/min, the measuring sequence duration reached up to 4 min. The regeneration sequence of the arterial blood to venous blood took between 15 and 30 min.

### 3.2. Testing the Effect of a Recirculation Loop on the Oxygen Transfer Rate

**Figure 4:**
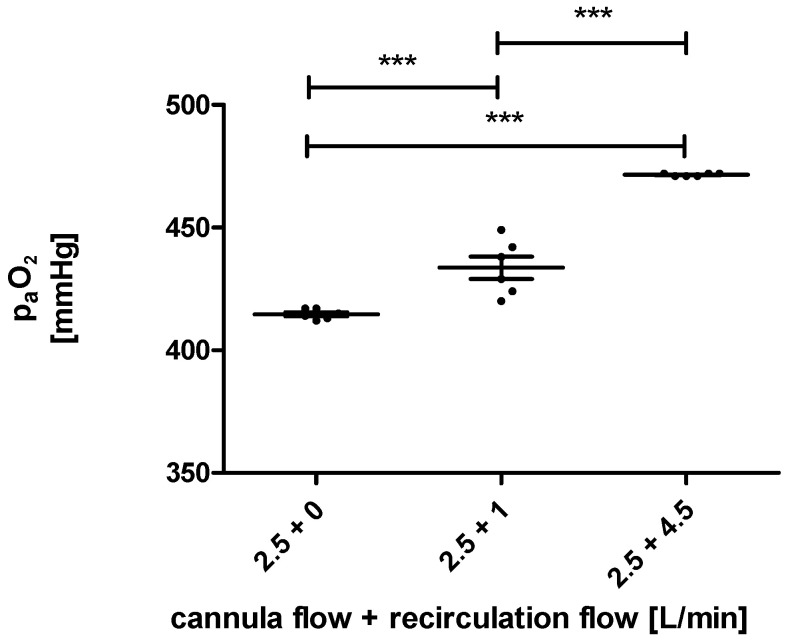
The measured oxygen pressure (p_a_O_2_) at the arterial sensor of the ECMO for a cannula blood flow of 2.5 L/min and blood flows over the recirculation loop of 0 L/min, 1 L/min, and 2.5 L/min are highly significantly different (***).

As shown in Figure 4, the oxygen pressure at the arterial sensor in the model was significantly different depending on the flow over the recirculation loop. The setup with no recirculation flow had the lowest oxygen pressure. Opening the recirculation loop led to increased oxygen pressure. The most potent effect was detected for the highest recirculation flow of 4.5 L/min. Under these conditions, the membrane lung received mixed blood instead of venous blood as input.

In addition, the recirculation loop also caused a change in the fluid dynamics of the ECMO setup. The pump of the ECMO in the two setups with recirculation required higher rotations per minute (rpm) in order to keep the cannula flow at 2.5 L/min. The setup with a closed recirculation loop needed 2055 rpm to generate 2.5 L/min of cannula flow, while the setup with a 1 L recirculation flow required 2225 rpm, and the setup with 4.5 L/min recirculation flow required 3060 rpm for 2.5 L/min of cannula flow.

**Figure 5:**
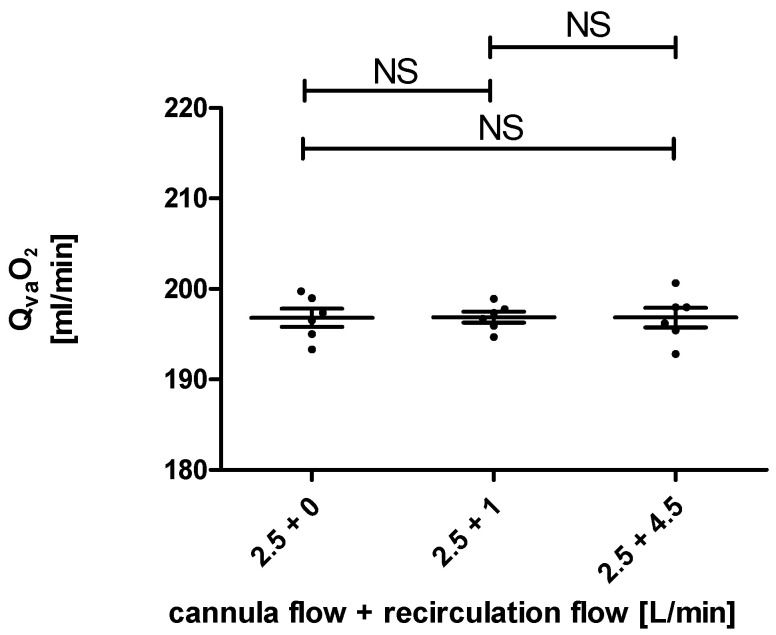
The calculated oxygen transfer rates over the membrane lung (Q_va_O_2_) for a cannula blood flow of 2.5 L/min and blood flows over the recirculation loop of 0 L/min, 1 L/min, and 2.5 L/min are not significantly different (NS).

Finally, the recirculation loop did not change the oxygen transfer rates. As shown in Figure 5, they were not significantly different between the measurements with a recirculation flow of 0 L/min, 1 L/min, and 2.5 L/min. In all measurements, oxygen transfer rates over the membrane lung in the range of 190 mL/min to 200 mL/min were observed.

## 4. Discussion

In this work, we presented a novel approach for an ECMO mock that can measure oxygen transfer at blood flow rates higher than 1.5 L/min. The main findings were that the presented mock circuit can be used for complex experiments, and can generate results that are comparable to animal-based research. Moreover, using the mock, we were able to gain a better understanding of recirculation loops and how they affect oxygen transfer efficiency.

### 4.1. Design of Our ECMO Mock Model

A major problem of mocks with high blood flow is the regeneration of the blood back to its venous condition. When we considered the design of this full-flow-capable ECMO mock, two solutions came to mind. The first solution was to use a higher number of membrane lungs for deoxygenation. If one membrane lung with N_2_ sweep gas was unable to remove the oxygen of one membrane lung with O_2_ sweep gas, perhaps multiple membrane lungs with N_2_ sweep gas in a series would do the job. However, we estimated that it required probably more than 10 membrane lungs with N_2_ sweep gas just to compensate for one normal membrane lung with O_2_ at full flow. As a result, that option was dismissed due to the high cost of membranes and the huge amount of N_2_ and CO_2_ gas necessary to run the tests. Instead, we chose a different approach. By performing the measurements and the venous regeneration discontinuously, the length of time required for deoxygenation vs. oxygenation was no longer an issue. In fact, as a bonus, the discontinuous design required only one membrane lung that could be used alternatingly with O_2_ sweep gas for testing, and with N_2_ and CO_2_ sweep gas for blood regeneration. On the other hand, and in contrast to our previous ECCO_2_R mock, the new ECMO mock is functionally a single-passage model during the measuring sequence; this also causes some limitations.

#### 4.1.1. Limitations of Our Mock Model

During each measuring sequence, the available blood volume is pumped from bucket A to bucket B until bucket A is empty. Therefore, the time of each measurement series is limited both by the amount of blood in bucket A and the blood flow of the system. The buckets we used can take 12 L of pig blood, allowing us to perform measurement series of around 2–3 min, which is enough time to collect reasonable data. The relatively high volume of porcine blood required was also not an issue for us because those quantities are routinely available as waste that would otherwise be discarded at the local butchery.

The long regeneration time after each measurement represents the second limitation. The deoxygenation of 12 L of 100% saturated blood back to a saturation of 65% routinely takes 30 min with an N_2_ sweep flow of 8.5 L/min.

Moreover, our model in its current form does not feature any physical cannulas. Therefore, the effect of any cannula-related aspect—for example, the comparison of the oxygen intake of veno-arterial (VA) and veno-venous (VV) ECMO configurations—cannot be simulated by our model.

#### 4.1.2. Comparison of Our ECMO Mock Model to Other Mock Models

The idea of reducing animal models and promoting alternatives for animal-based experiments is very popular in the scientific community and politics [12]. For ECCO_2_R, the establishment of a mock circuit shows a reasonable alternative to animal models. For ECMO, only a few other mock circuits are already described in the literature [4,6,7,8,9]. Most of them investigate parameters such as coagulation and obstructions in the whole ECMO system [6,8]. Bleilevens et al. established an in vitro mock circuit that operates feasibly under physiological conditions for more than 6 h [4]. However, blood flow of the operating system was only set at 0.6 L/min, and oxygen transfer, which defines the efficiency of the membrane lung, was not measured. As far as we know, a mock circuit that can investigate the efficiency of a membrane lung applying different blood flow rates while varying other parameters has not been described before. Therefore, our setup is a pioneer in the investigation of feasible alternatives for animal-based experiments. The oxygen transfer rates we obtained for a blood flow rate of 2.5 L/min without recirculation are in the same range as published results using an animal model with pigs. Therefore, we are confident that the oxygen transfer rates measured with our mock are valid [16].

### 4.2. Testing the Effect of a Recirculation Loop on the Oxygen Transfer Rate

The second aim of this work was to investigate recirculation loops. Recirculation loops are supposed to increase both the oxygenation and CO_2_ removal ability of ECCO_2_R and ECMO setups [17]. So far, we were able to show the effect of a recirculation loop on CO_2_ removal in our ECCO_2_R model; however, until now, we did not have an appropriate model to investigate the effect on oxygenation [12].

As pointed out by Madhani et al., the expected improved oxygenation when using a recirculation loop is the result of a reduced boundary layer on gas fibers due to increased blood velocity in the membrane lung [17]. This boundary layer is a stationary fluid layer that forms on gas fibers and impedes oxygen transfer.

Our results reflected this expected outcome, as the post-membrane p_a_O_2_ rose with an increased blood flow in the recirculation loop. However, the increase in p_a_O_2_ values did not translate to significantly higher oxygen transfer rates. The reason for this is that physically dissolved oxygen only accounts for a small part of the oxygen transfer in blood. The observed increases in paO_2_ translate to increases in the oxygen transfer rate of only 2 to 4 mL/min. Those changes are too small compared to the noise in oxygen transfer rate measurement, caused by small fluctuations in the blood flow.

Although the recirculation loops caused slight increases in oxygen transfer, they also added additional tubing and bends to the ECMO setup, which might increase the risk for coagulation and inflammatory pathways. However, recirculation loops have also been described as tools that reduce the risk of membrane clotting due to the higher blood velocity in the membrane lung [18]. All in all, those benefits and risks of recirculation loops need further investigation in future research.

## 5. Conclusions

This is the first study to present an ECMO mock circuit capable of measuring oxygen transfer rates at high blood flow rates. This opens new avenues for animal-free ECMO research. The model was successfully applied to better understand how recirculation loops effect ECMO physiology.

## Data Availability

Data can be provided upon request addressed to the corresponding author. All data-sharing statements are subject to conformity with German data protection legislation and rules (Datenschutzgrundverordnung—DGSVO).
